# Activation of Content-Schemata for Scaffolding L2 Writing: Voices from a Turkish Context

**DOI:** 10.1007/s10936-023-10002-3

**Published:** 2023-08-25

**Authors:** Elmaziye Özgür Küfi

**Affiliations:** 1 School of Education and Communication, Jönköping University, P.0 Box 1026, 55111 Jönköping, Sweden; 2 Foreign Languages and English Preparatory School, Eastern Mediterranean University, Via Mersin 10, Famagusta, Northern Cyprus Turkey

**Keywords:** Writing skill, L2 writing, Insufficient world knowledge, Content-schemata activation, Integrated-skills approach to writing

## Abstract

It has been observed that Turkish university students suffer in L2 writing when they lack background knowledge about the writing topic. Triggered by this observation, this study intended to explore effectiveness of content-schemata activation for scaffolding Turkish students in their challenging L2 writing practices. Study participants, students studying at an English-medium university in Turkey, were asked to write an essay on a specific topic at the beginning of the week before participating in any activities and then they were asked to write a second essay on the same topic after being engaged in various skills activities designed to activate their content-schemata. The same procedure was repeated for seven weeks with a different topic each week. To gather data, students’ first and second essays were compared and students’ and teachers’ perceptions regarding their experiences in English writing classes were elicited through questionnaires. As study findings reveal that content-schemata activation leads to the production of better essays in terms of content and that both students and teachers are positive about the use of skills activities for idea generation prior to essay writing, integration of activities that would activate students’ content-schemata into the language curriculum in other ESL/EFL educational contexts is highly recommended.

## Introduction

With the advent of Covid-19, writing has become a more valued skill globally since practitioners, no matter in which sector they work, have become highly dependent on written computer-mediated communication for effective exchange and dissemination of information in their fields (Wood & Schatschneider, 2021). Despite this increased worldwide significance of writing, L2 writing is still not a popular activity for ESL/EFL students in some educational contexts as they feel anxious and challenged when asked to do a writing task in English (Alisha et al., 2019; Anh, 2019; Faraj, 2015; Fareed et al., 2016; Ibnian, 2017; Qomariyah & Permana, 2016; Sabuncuoğlu, 2018; Selvaraj & Aziz, 2019). ESL/EFL learners’ writing difficulties get more prominent at tertiary-level as they are required to undertake various academic writing tasks in English, all of which have a huge impact on their overall academic performance (Graham & Perin, 2007; Tavşanlı et al., 2020). According to researchers, the difficulty of writing stems from the fact that it is a “complex, multidimensional construct” which involves coordination of cognitive, linguistic and emotional resources (Wood & Schatschneider, 2021, p. 2) as well as idea generation and organization, which some learners find particularly difficult due to lack of prior knowledge about the writing topic (Selvaraj & Aziz, 2019). These difficulties require ESL/EFL teachers to not only understand the reasons why writing activities are challenging for their students but also find ways of scaffolding their writing practices.

In the history of writing instruction, two approaches have been the most influential; the traditional approach which focuses on the product of writing and the process approach that puts emphasis on the process. The product view of teaching writing required ESL/EFL teachers to instruct certain principles based on ideal models, which students were expected to imitate while writing their essays in a limited period of time. Even in the early days, this traditional approach was criticised for creating “an unproductive and inappropriate orientation toward composition” (Chastain, 1976, p. 252) and its impact on students’ writing practices was believed to be negative. Students’ having a writer’s block, writing anxiety and the use of ineffective composing strategies were some negative effects of the product-based approach reported in those days (Zamel, 1987). As more and more research provided insights into its negative effects, a gradual shift to process-oriented pedagogy was observed in writing classes. Unlike the traditional approach, in the process approach writing is perceived as a non-linear process that involves consideration of the audience, the purpose of writing, generation and organization of ideas as well as production of several drafts.

In light of recent developments in language education, ESL/EFL teachers have abandoned writing practices which required students to write a complete essay in one sitting and started teaching writing as a recursive process and a means of communication in the language classroom (Bayat, 2014; Faraj, 2015; Graham & Sandmel, 2011; Sabuncuoğlu, 2018). Nowadays, language teachers view writing as a creative activity and are aware of the fact that students need guidance not only with the linguistic aspects of writing but also in producing the content of their essays (Fareed et al., 2016; Ibnian, 2017; Selvaraj & Aziz, 2019; Tavşanlı et al., 2020) Although there is abundant research on writing pedagogies (Li et al., 2022), studies that specifically focus on idea generation or activation are scarce (Jouhar & Rupley, 2021; Wood & Schatschneider, 2021; Zarrinabadi & Rahimi, 2021). To fill this research gap, there is a need for studies that will yield evidence as to how scaffolding can be provided in L2 writing classes when students suffer from lack of background knowledge on the writing topic.

## Schema Theory

Schema theory, which has been mainly investigated in the reading field, asserts that a text does not carry meaning by itself; it only provides directions as to how readers should retrieve meaning from a text by using their prior knowledge (Carell & Eisterhold, 1988; Mahmood et al., 2013; Manzo & Manzo, 2013). In other words, the schema theory explicates that comprehension takes place if the reader is able to bridge between his/her prior knowledge and what is presented in a reading passage. Sometimes students state that they do not understand a paragraph although they know every word in it. Students’ lack of comprehension in such cases can be explained through schema theory which “hypothesises that knowledge is stored in the mind in abstract scaffolds or frameworks called schemata” and modified based on experiences over the course of time (Aron, 1986, p. 11).

Prior to the schema theory, characteristics of the reader and the contexts in which reading occurred were completely ignored while the text and the skills necessary to understand it received great emphasis (Norris & Phillips, 1987). With the schema theory, educators have realized that knowledge is stored in ‘packets of knowledge’ called schemata and that ‘meaning’ cannot be considered independent of the reader (Perkins & Angelis, 1985, p. 269). Studies on schema theory have also enlightened educators about the fact that communication relies not only on the linguistic knowledge but also the interactions among the reader, the reading context, and the text. While exploring how prior knowledge affects reading comprehension, researchers have drawn a distinction between formal and content-schemata (Maftoon & Babamiri, 2011) and explained formal schemata as “background knowledge of formal, rhetorical organizational structures of different types of texts” and content-schemata as “background knowledge about the content area of a text” (Carell & Eisterhold, 1988, p. 80).

Researchers contend that activating ‘semantic memory’, another term used for content-schemata, is an effective pedagogical device that prepares students for reading as it “triggers off a series of associations, and all the connotations are activated and brought, as it were, to the front of the mind” (Statman, 1981, p. 232). They explain that in this way students can develop expectations of what is to come, and they do not feel that the subject is completely new. It is also reported that ‘semantic memory’, knowledge that develops in the brain when a meaning-loaded term is encountered, is limited in the working memory of students with low proficiency, which makes activation of their world knowledge difficult (Jouhar & Rupley, 2021). Hence, schemata activation is particularly suggested for these students to enable them to build new concepts and have a sense of direction and security in the learning process. As research findings indicate that students’ reading can be improved when they are helped to build background knowledge on the topic prior to reading (Taglieber et al., 1988), engaging students in activities that activate their content-schemata before reading tasks has long become a common practice in L2 classrooms. Despite the focus on reading skills in language research and acknowledgement of additional reading support needs of students who come from low socio-economic backgrounds due to lack of technological resources during the pandemic (Sucena et al., 2022), the impact of content-schemata activation on students’ writing skills is still an under-researched area.

## L2 Writing and Pre-writing Activities

As writing in ESL/EFL educational settings is part of learning a foreign language, students face a set of linguistic difficulties such as using tenses incorrectly, missing out articles or not finding the right words to express their thoughts depending on their language proficiency level. Another problem ESL/EFL students have in L2 writing is finding what to write. Sometimes students cannot generate ideas due to insufficient knowledge about the topic of the writing task and hence they end up with essays empty of original ideas. The lack of knowledge about essay topics also causes students to feel anxious during writing lessons and to dislike L2 writing (Bayat, 2014; Ibnian, 2017). Since university students are expected to hand in well written term papers and reports or to provide coherent responses to essay-type examination questions in tertiary-level education systems, their negative attitudes towards L2 writing generally affect their overall academic performance negatively (Tahmouresi & Papi, 2021).

With recent studies on process approach, language teachers started to attach importance to the stages of writing such as generating, formulating, refining ideas, drafting, and revising (Faraj, 2015; Graham & Sandmel, 2011; Selvaraj & Aziz, 2019). Realization of how significant idea generation is in L2 writing has perhaps been the most crucial outcome of research as it has led scholars to put more emphasis on pre-writing activities, which are defined as “any structural experiences that influence active student participation in thinking, talking, writing and working on the topic under focus in a writing lesson” (Oluwadiya, 1992, p. 12).

Different kinds of pre-writing activities may be used to help learners acquire new knowledge and skills or build on what they already know (Sabuncuoğlu, 2018). Use of skills activities at the pre-writing stage may bring students’ prior knowledge into a form that can be used in their writings enabling them to acquire new information and add to their existing knowledge. While engaging students in activities that will generate their ideas, it is necessary to help them to connect their own experiences to the world outside. To this end, recent studies suggest integration of a variety of activities into the language classroom for improving language learners’ knowledge regarding their disciplines and structural analysis (Li et al., 2022) and particularly all four language skills based on the rationale that “the four language skills support each other and are found together in real-life language use” (Sabuncuoğlu, 2018, p. 128).

Primarily, reading and writing activities are suggested for the pre-writing stage as they are mutually reinforcing processes. Researchers note that development of students’ reading strategies result in the improvement of their writing as well because the process of reading involves both extraction and the supply of information (Jouhar & Rupley, 2021; Sabuncuoğlu, 2018). Reading activities also encourage students to think critically as it engages them in “logical reasoning, independent thinking and careful analysis of text” (Spack, 1985, p. 721; Jouhar & Rupley, 2021). Integration of reading skills is therefore suggested for activating ESL/EFL students’ content-schemata and expression of ideas in a meaningful way in their L2 writing practices (Jouhar & Rupley, 2021).

Listening tasks can also be used as authentic activities in writing lessons because in real life people note down ideas and then re-use them in another form (Byrne, 1988). The use of listening activities before L2 writing can be beneficial especially for ESL/EFL students who study at universities where the medium of instruction is English as they need to use both skills to participate in lectures, seminars or discussions. Listening activities are also useful for activating ESL/EFL students’ content-schemata on the writing topic because to understand the fundamental ideas expressed in spoken discourse, they need to refer to their world knowledge and prior experiences. Similarly, in speaking activities students depend on their world knowledge and prior experiences to a great extent because this is the main source for them to express their ideas. While conveying and receiving information in speaking activities, students’ content-schemata are naturally activated because they continuously refer to their knowledge and experiences in order to keep the conversation going on. Writing activities also provide a means for getting students to think, write and work on a topic about which they will write later. These activities may involve brainstorming, clustering, looping, outlining, or using graphic organizers and formative assessment as suggested by recent research (Mahmood et al., 2013; Tavşanlı et al., 2020).

As the current university student generation has grown up in digital environments with the internet, integration of computer-mediated learning platforms and online forms of communication into their writing experiences is a logical pedagogical approach to take in tertiary level language education (Thorne & Reinhardt, 2008). However, as cautioned by researchers, limitations of technological tools such as lack of personalization and emotions, which play a significant role in students’ written performance, need to be considered (Shahriar & Hayawi, 2023; Zhang & Hyland, 2018). In brief, the use of pre-writing activities, whether it is a reading, listening, speaking, or writing task in digital or non-digital format, enables ESL/EFL students to think about the content of their essays in advance by establishing a connection between their background knowledge, their prior experiences, and the topic at hand. While thinking about the content, students’ content-schemata are activated because they strive to better understand, analyse, or view the issue from a different perspective. In other words, these activities help students to uncover what they already know and think. Thus, as suggested in a relevant study, engaging students in language activities that get them to use all four skills can enable the creation of real-life like situations in the language classroom (Sabuncuoğlu, 2018).

## Methodology

Considering the significance of writing for Turkish students in tertiary level education and particularly the students who suffer in L2 writing due to lack of sufficient world knowledge, the present study made use of skills activities to activate Turkish ESL/EFL students’ content-schemata and intended to find out whether content-schemata activation has an effect, if any, on their L2 writing practices.The study had a sequentially exploratory mixed method research design as it employed the use of quantitative and qualitative methodology in an integrated way in successive stages (Ivankova et al., 2006). The former was used to test the following hypothesis: When students are exposed to skills activities that activate their content-schemata, they will write better compositions—in terms of content—than when they are not, and the latter was employed to elicit students’ and teachers’ perceptions about their experiences regarding the use of skills activities for the enhancement of students’ writing skills. To guide qualitative data collection and analysis processes, the following research questions were posed:

- How did the participating students perceive the use of skills activities for activating their content-schemata prior to essay writing?
- How did the participating teachers perceive the use of skills activities for activating their students’ content-schemata and improving their writing skills?

## Data Collection

The present study was conducted in a class of the foundation school at an English-medium university in Turkey. Participants were students studying in the upper-intermediate (B2) level of the Intensive English programme. In this programme, an in-house course-book, designed by the curriculum team of the school, was used as teaching material. Participating students, who were between 18–20 years old, were provided the same time and conditions throughout the study. 14 students could participate in the study because the school management determined the maximum class size for B2 level classes as 14. To gather data, students first and second essays were evaluated, and student and teacher questionnaires were employed to elicit perceptions. All the participants were informed about the research purpose, procedures and their informed consent was secured prior to the implementation of the study.

### Student Essays

The students were asked to write their first compositions at the beginning of the week before being engaged in any activities. To ensure and maintain the same conditions for all students, the researcher asked them to write their compositions in class without using any sources and the time for writing the first composition was limited to fifty minutes. The activities used for activating students’ content-schemata prior to writing their second composition included all four language skills either in an integrated way or separately. To elaborate, students read passages about the weekly topic, listened to recorded texts and took notes, spoke about the topic in class discussions, created mind maps in brainstorming activities and took part in many other activities. Each week students were engaged in skills activities about a different topic and afterwards they were asked to write the second composition of the week. The principles of process approach were used to guide students to write their second compositions. Students wrote their first drafts in class, and they were again given fifty minutes to write it. In this way, the influence of outside variables like plagiarism or getting someone else’s help could be avoided.

After considering the writing criteria used in the language school in the context of the study as well as another scale used in the English preparatory school of a well-established university in Turkey, a scale was designed for evaluating student compositions (Please see Appendix A). As the evaluation scales used in these language institutions focused on every aspect of a composition (content, grammar, vocabulary, and organization), the researcher felt the need to design a specific scale for evaluating students’ first and second compositions mainly in terms of ideas. Designing an original set of criteria for the purposes of this study was an intricate process since every sentence or even a word written by a student could be representing an idea. However, considering that counting every sentence or word written by a student would not reveal reliable results for an academic study, writing tasks assigned to the students in the research context were analysed before the creation of a specific evaluation scale for the purposes of this study.

The evaluation scale was employed every week to determine whether students’ first and second compositions differed in terms of ideas. After evaluating both compositions, the scores students got from their first and second composition were compared to find out the difference between the two compositions in terms of content. For the organization criterion in the checklist, students’ ideas in the development paragraphs were evaluated in terms of the main idea’s relevance to the thesis statement and the successful use of supporting ideas to develop it. In the conclusion paragraph, the expression of students’ personal comment was the main criterion. As students were exposed to many words related to the composition topic via the skills activities, it was presumed that the appropriate use of these words would indirectly show the improvement of ideas. Thus, based on the idea that clear expression of ideas can be maintained by appropriate choice of vocabulary related to the topic, vocabulary was added as a component to the evaluation scale and to reward students who had original ideas in the essay with some extra points, the content component was also integrated into the evaluation criteria.

Students’ compositions were evaluated by two teachers, one of whom was the researcher. For the purposes of anonymity and confidentiality, students’ names were not disclosed and the papers which were assigned different grades by the evaluators were moderated to reach an agreement. To ensure the two evaluators’ objectivity and inter-rater reliability, allocated scores were cross-checked by three other teachers who randomly selected and read three pairs of compositions written by the students each week.

### Questionnaires

Questionnaires were designed to elicit students’ and teachers’ perceptions about the writing skill in general and the use of skills activities before writing an essay. Prior to the design of the questionnaires, several sample instruments used at language schools were considered. However, as the study was a novel in terms of the activation of content-schemata in L2 writing, the questions were originally written by the researcher of the study in accordance with the research design. To ensure reliability of each questionnaire, an interactive approach was adopted during the questionnaire administration. In this way, participating students and teachers were able to raise questions about instructions and/or content of the questionnaire when needed. This provided a constructive context for the provision of consistent feedback to all participants by the researcher, relevant responses by the participants, and overall reliability of the questionnaires.

As it can be seen in Appendix B, the student questionnaire consisted of 25 items, five of which aimed to explore students’ awareness of the writing skill while the remaining ones intended to elicit students’ ideas related to the ‘content’ of their compositions. The teacher questionnaire consisted of 11 structured questions (please see Appendix C). Open-ended questions were not included in the questionnaire to minimize evasive answers that would cause analysis problems and to save on time. The student questionnaire was administered at the end of 7 weeks, that is, after students had written seven first compositions, been exposed to various skills activities and written seven second compositions. Both questionnaires were administered at the end of 7 weeks and teacher respondents were fourteen teachers who had taught B2 level students and used similar skills activities and writing tasks in their classes.

## Results and Discussion

### Student Essays

Student compositions were analysed using a reliability test and a paired samples T-test. 150 papers were collected in total. Each essay was evaluated by two scorers. The Pearson Correlation Coefficient Test was used to check the reliability of the evaluators’ scores. The correlation coefficient, shown by **r** (Pearson coefficient) in Table 1, ranges in value from -1 to + 1 and 0 indicates that there is no relationship between the two variables. The significance level, shown by **p** (two tailed significance) in Table 1, was set as *p* < 0.05. Table 1 displays that the evaluators’ scores were almost perfectly related since the correlation is very close to + 1 and the two tailed significance is smaller than 0.05 in all cases except Week 2.

**Table 1:** Results of the reliability test for the evaluators’ scores

		First essay	Second essay
Week 1	r	0.8140	0.9479
11 Students	p	0.002	0.000
Week 2	r	0.9642	0.7040
8 Students	p	0.000	0.0051
Week 3	r	0.9071	0.8762
11 Students	p	0.000	0.000
Week 4	r	0.9953	0.9723
9 Students	p	0.000	0.000
Week 5	r	0.9039	0.9614
14 Students	p	0.000	0.000
Week 6	r	0.9787	0.9031
11 Students	p	0.000	0.000
Week 7	r	0.9023	0.9738
11 Students	p	0.000	0.000

Table 1 shows an exact correspondence between evaluator scores in 20% of the papers. There was 1 grade difference in 30.33% of the papers. In these papers, the higher grade was considered. There was a 2-grade difference in 24% of the papers. In these papers, the middle score was taken into consideration. There was a greater discrepancy between the remaining 26.6% papers. These grades were computed without making any changes to ensure effective application of the correlation test. However, for the paired samples T-test, these papers were re-read and discussed by the evaluators until an agreement, which included a maximum of 2 grade difference, was reached.

### T-Test Results

Paired Samples T-test was used to compare the scores of the first compositions and the second compositions on a weekly basis. These results are displayed in tables on a weekly basis.

The composition topic of week 1 was “How to be an efficient learner”. Three students were absent when the compositions were written. As it can be seen from Table 2, there is a significant mean difference between Composition 1 and Composition 2 (0.002 < 0.05), which reveals that there was enrichment of ideas in the students’ second compositions in the first week.

**Table 2:** Paired samples T-test results of week 1 compositions scores

Variable	Number of pairs	Corr	2-tail Sig	Mean	SD	SE of mean
COMP1				7.2727	3.165	.954
	11	.968	.017			
COMP2				12.0909	5.281	1.592

Mean	SD	SE of mean	t-value	df	2-tail Sig
*Paired differences*					
− 4.8182	3.816	1.151	− 4.19	10	.002
95% CI (− 7.383, − 2.254)					

The composition topic of week 2 was “The Differences between Men and Women”. Only 8 students wrote both compositions. Some of the students were absent while others participated in another school activity. As Table 3 displays the mean difference between Composition 1 and 2 in week 2 is 0.011 < 0.05. Hence, it can be stated that there is an improvement in the content aspect of students’ compositions in week 2.

**Table 3:** Paired samples T-test results of week 2 compositions scores

Variable	Number of pairs	Corr	2-tail Sig	Mean	SD	SE of mean
COMP1				9.6250	5.397	1.908
	8	.130	.759			
COMP2				16.3750	2.264	.800

Mean	SD	SE of mean	t-value	df	2-tail Sig
*Paired differences*					
− 6.7500	5.574	1.971	− 3.43	7	.011
95% CI (− 11.411, − 2.089)					

The composition topic of week 3 was “Many species are becoming endangered. Why is this and what can be done to protect them?”. 11 students wrote both compositions. As it can be seen from Table 4, the mean difference obtained this week is again significant since the p level is 0.000.

**Table 4:** Paired samples T-test results of week 3 compositions scores

Variable	Number of pairs	Corr	2-tail Sig	Mean	SD	SE of mean
COMP1				6.0000	3.606	1.087
	11	.395	.229			
COMP2				12.5455	2.806	.846

Mean	SD	SE of mean	t-value	df	2-tail Sig
*Paired differences*					
− 6.5455	3.588	1.082	− 6.05	10	.000
95% CI (− 8.956, − 4.134)					

The composition topic of week 4 was “The role of tourism in your country”. Nine students wrote both compositions of week 4 and as can be seen in Table 5, the mean difference in week 4 is again quite significant since it is 0.001.

**Table 5:** Paired samples T-test results of week 4 compositions scores

Variable	Number of pairs	Corr	2-tail Sig	Mean	SD	SE of mean
COMP1				3.6667	3.202	1.067
	9	.471	.201			
COMP2				11.2222	4.868	1.623

Mean	SD	SE of mean	t-value	df	2-tail Sig
*Paired differences*					
− 7.5556	4.391	1.464	− 5.16	8	.001
95% CI (− 10.931, − 4.180)					

In week 5, students were given the following instruction before writing their compositions: “Government is considering banning TV for one or two days per week. Write a letter to a local newspaper stating your opinion”. All 14 students wrote both of the compositions. Table 6 shows that the results are again significant since the mean difference is 0.000.

**Table 6:** Paired Samples T-test results of week 5 compositions scores

Variable	Number of pairs	Corr	2-tail Sig	Mean	SD	SE of mean
COMP1				4.5714	2.277	.609
	14	.424	.131			
COMP2				11.1429	4.688	1.253

Mean	SD	SE of mean	t-value	df	2-tail Sig
*Paired differences*					
− 6.5714	4.256	1.137	− 5.78	13	.000
95% CI (− 9.029, − 4.114)					

In week 6, students were asked to design a form of transport for the future. A lot of students in class refused to write the first composition. Some students stated that they didn’t have any ideas to write about on this topic and others shared that they felt overwhelmed because they had an exam on that day. Although the teacher, also the researcher of this study, insisted on getting a first composition from the students, she realized that some students had only written the introductory paragraph of their first composition or left the paper blank. Because of this reason, most of the first compositions were given a score of ‘0’, which explains the reason why the mean difference shown in Table 7 is 0.000. Despite the problems faced in writing the first composition in this week, it can be claimed that students benefited from skills activities done before the second composition because some of them reported that they couldn’t write their first compositions due to insufficient knowledge about the topic.

**Table 7:** Paired samples T-test results of week 6 compositions scores

Variable	Number of pairs	Corr	2-tail Sig	Mean	SD	SE of mean
COMP1				1.8182	3.157	.952
	11	.502	.116			
COMP2				12.3636	3.075	.927

Mean	SD	SE of mean	t-value	df	2-tail Sig
*Paired differences*					
− 10.5455	3.110	.938	− 11.25	10	.000
95% CI (− 12.635, − 8.156)					

The composition topic of week 7 was “Food”. Students were dictated five statements about food and they were asked to write their first and second compositions about the statement they most agreed with from the given statements. As it can be seen from Table 8, there was a significant mean difference (0.000 < 0.05) between composition 1 and composition 2 in this week as well.

**Table 8:** Paired samples T-test results of week 7 compositions scores

Variable	Number of pairs	Corr	2-tail Sig	Mean	SD	SE of mean
COMP1				2.1818	2.228	.672
	11	.433	.183			
COMP2				9.3636	2.976	.897

Mean	SD	SE of mean	t-value	df	2-tail Sig
*Paired differences*					
− 7.1818	4.423	1.334	− 5.39	10	.000
95% CI (− 10.154, − 4.210)					

Overall, T-test results showed that there was a significant difference between the scores of students’ first and second composition in each week which can be attributed to the use of skills activities which activated students’ content-schemata and led to the enrichment of ideas in their second compositions.

### Questionnaires

Quantitative data collected from questionnaires were analyzed using the SPSS (Statistical Package for Social Sciences) programme. After the analysis of quantitative data, questionnaire results were interpreted considering participants’ perceptions about their experiences in the writing class and content-schemata activation via skills activities prior to essay writing. To this end, qualitative data which consisted of participants’ opinions and feelings were content analysed and emerging themes were highlighted.

### Student Questionnaires

Student responses revealed that all students thought of writing as an important skill. Half of the students said that writing was important for them because they had to do notetaking from different sources and a minority stated that they needed to use the skill of writing for answering essay-type examination questions. Many students reported that they did not enjoy writing activities in their course-book because they found them boring. Interestingly however, students noted that writing topics were contemporary as they were about daily life, environmental or cultural issues. Almost half of the students (42.9%) shared that ‘content’ is the most important component of a composition. This finding is in line with results of the paired samples T-test and the assumptions made in the study since the majority of students (71.4%) mentioned that they prefer their teacher to support them via skills activities before a writing task. This was confirmed by 78.6% of the students who stated that the teacher’s attitude during the first composition was demoralizing as she did not give any guidance. Furthermore, 64.3% of the students reported that the support provided by the teacher prior to the writing process of the second composition caused them to enjoy writing classes and increased their motivation to improve their writing.

A great majority of the students (92.9%) stated that the first composition was more difficult to write and most of them (71.4%) attributed this difficulty to their insufficient knowledge about writing topics. They explained that they could acquire necessary knowledge about the writing topic because of the activities they did in class prior to writing the second composition. 7.1% of the students noted that these activities increased their knowledge by a hundred percent, 14% said that they increased their knowledge by seventy-five percent, 50% stated that they increased their knowledge by fifty percent and 28% reported that they increased their knowledge by thirty percent. None of the students chose the 0% option which denied the influence of the activities on the improvement of their knowledge regarding the essay topic. Analysis of students’ responses also revealed that more than half of the students found reading activities done prior to second compositions beneficial. A vast majority of students stated that they prefer the use of reading activities before writing compositions.

Despite their positive perceptions about reading activities, students reported that they didn’t benefit from the listening activities. Students reported that they were content with speaking activities because participation in speaking activities enabled them to exchange ideas and share information with their friends and the teacher. A great majority said that speaking activities should be used before writing tasks while only a minority reported that the speaking activities should not be done at all. Likewise, most of the students reported that writing activities were beneficial and should be used before writing compositions because they give them the opportunity of combining their own ideas with what is done in class. Some students underlined the fact that writing will be even more important for them when they start taking their departmental courses. A small minority, on the other hand, reported that they did not believe in the benefit of writing activities because they thought that these are the kind of activities that can be done better at home than in class and also because they did not find the writing topics interesting.

Overall, the results obtained from the student questionnaires showed participating students’ awareness of the significance of L2 writing in tertiary-level education and the gaps in their knowledge regarding the writing topics. Student perceptions also revealed that they benefited from the use of skills activities as they reported that they felt more competent while writing the second composition of the week after participating in various skills activities. Related to their experiences about L2 writing in general, some of them even shared that they started to enjoy essay writing in English.

### Teacher Questionnaires

Parallel to student responses, teachers reported that most of the students in their class did not enjoy writing. The majority of the teachers (71.4%) explained the underlying reason for their students’ dislike of writing as students’ feeling indifferent, some getting anxious when given a writing task and some feeling bored. Similarly, recent research reveals that there can be differential growth in the writing of students who come from different backgrounds (Wood & Schatschneider, 2021). In contrast to students’ dislike of the writing activities in the course-book*,* the majority of the teachers (71.4%) thought that the writing tasks in the course-book were much better than the writing activities in commercial textbooks. Students’ and teachers’ different perceptions related to the writing activities in the in-house course-book used in the B2 level programme can be related to the use of the process approach, which required students to invest more time and effort for the writing tasks. The discrepancy between students’ and teachers’ ideas can also be seen in research as one study reports that the process approach does not improve students’ writing performance (Graham & Sandmel, 2011) whereas others discuss its’ positive influence on students’ writing (Selvaraj & Aziz, 2019).

Majority of the teachers (92.9%) reported that they prefer using different kinds of activities in their writing classes prior to essay writing. Since both the teachers and students are positive about the use of skills activities before writing tasks, different skills activities should be used to help students generate ideas as suggested in the literature (Ibnian, 2017; Sabuncuoğlu, 2018). Teachers’ and students’ perceptions of the ‘idea’ component of a composition were also parallel since all the teachers reported that they prefer a composition with some grammar mistakes or organization problems yet rich in ideas to a well-organized composition with very few grammar mistakes but poor in ideas. Participating teachers noted that their students struggled with writing because they did not possess sufficient world knowledge about the writing topics. Related to this difficulty, a great majority (85.7%) reported that this gap in students’ knowledge can be filled by using skills activities that activate their content-schemata. This finding is in line with research which suggests integration of all four skills into the language class to help students acquire new knowledge and skills or build on what they already know (Faraj, 2015; Sabuncuoğlu, 2018).

The great majority of the teachers noted that all types of skills activities are useful for helping students to write their compositions as they facilitate acquisition of information about the writing topic by providing background knowledge. This finding is in line with recent literature which discusses that teaching of writing should be harmonized with teaching of reading, listening and speaking skills (Anh, 2019; Ibnian, 2017; Sabuncuoğlu, 2018). Regarding the effectiveness of reading skill activities, students’ and teachers’ responses were parallel. A great majority of the students asked for the use of reading activities prior to writing tasks. Similarly, almost all of the teachers (92.9%) reported that reading activities should be used on a large scale because they are useful for not only expanding students’ knowledge about the writing topic but also for learning about the writing style and organization of different kinds of genres as discussed in the literature (Anh, 2019; Jouhar & Rupley, 2021).

Related to listening activities, some teachers mentioned voice quality of the recordings to be a problem. Like students, teachers noted that listening activities were less useful than reading activities in terms of scaffolding students’ writing. Study results in this respect are in line with research which calls for the use of different skills in an integrated way rather than separately (Anh, 2019; Faraj, 2015; Ibnian, 2017; Sabuncuoğlu, 2018). Parallel to student perceptions, a great majority of the teachers (92.3%) stated that speaking activities should be used on a large scale. Teachers explained their positive views about speaking activities by saying that during speaking activities students can express their ideas in a short time, which is helpful in stimulating their interests and activating their ideas or related vocabulary. These teacher beliefs are in accordance with the advocates of teaching pedagogies that aim to reduce anxiety and increase student motivation in the writing class (Bayat, 2014; Tahmouresi & Papi, 2021; Tavşanlı et al., 2020).

In the study, a great majority of teachers (89.2%) noted that writing activities should be employed before assigning an essay topic to the students because students need to consider writing activities as a natural part of learning a language rather than an occasional chore. A few teachers (7.7%) stated that they were against the use of pre-writing activities because they thought that students can use other means to collect information on a topic and that students can get bored when they are asked to do too many writing tasks. Related to this concern, consideration of methodologies that enhance students’ motivation and their perception of themselves as successful L2 writers can be a good idea as suggested in a recent research study (Tahmouresi & Papi, 2021).

Analysis of all the responses given to the questionnaire items reveals that both the students and the teachers found the use of reading and speaking activities more beneficial than listening and writing skills activities. The fact that students’ perceptions of skills activities were highly positive indicates that they were aware of the benefits of these activities for improving their writing performance and were also satisfied with the support these activities provided for idea generation. In this regard, findings of this study are parallel with the positive results of a recent study which revealed that the reading skills of students were significantly improved after a 5-week period of additional support on reading skills (Sucena et al., 2022). As the use of the process approach and skills activities that activate their content-schemata can provide scaffolding for students in L2 writing, ESL/EFL teachers should consider making use of both in their writing classes as recommended in relevant literature (Selvaraj & Aziz, 2019). In this regard, language instructors can benefit from AI tools like ChatGBT, however, while acknowledging the potential of most recent technological tools, they should be aware of their limitations like lack of personalization and emotions (Shahriar & Hayawi, 2023) as they can influence students’ attitudes, interest, attention, persistence, and engagement in writing tasks, all of which contribute to their writing endeavours (Zhang & Hyland, 2018).

## Conclusion and Recommendations

Although plenty of research studies have been conducted on the “effectiveness of intensive language programs” (Li et al., 2022, p.11), a considerable number of ESL/EFL students who study in these programmes still find L2 writing challenging (Alisha et al., 2019; Anh, 2019; Faraj, 2015; Fareed et al., 2016; Ibnian, 2017; Qomariyah & Permana, 2016; Sabuncuoğlu, 2018; Selvaraj & Aziz, 2019). Considering the scarcity of research on ESL/EFL students’ writing problems due to their insufficient knowledge about L2 writing topics (Bayat, 2014; Ibnian, 2017; Selvaraj & Aziz, 2019), the present study intended to investigate the use of content-schemata activation as a means for scaffolding ESL/EFL students in their challenging L2 writing practices. Similar to the positive outcomes of a research study on the activation of formal schemata for the improvement of students’ writing (Maftoon & Babamiri, 2011), the present study yielded promising results for the improvement of ESL/EFL students’ writing skills by disclosing that content-schemata activation leads to the enrichment of ideas in ESL/EFL students’ essays.

In addition to positive statistical analysis results of quantitative data, qualitative data obtained from participating students and teachers indicate that skills activities have a positive impact on students’ writing performance and that both students and teachers find the use of skills activities for the activation of students’ content-schemata prior to L2 writing beneficial. Based on the positive results of this study and in light of recent research, it is recommended that content-schemata activation is considered as a complementary component of language teaching programmes for improved writing practices in ESL/EFL educational settings not only in the current pandemic era which we are in but also in the post-pandemic era since “impairments in reading and writing acquisition skills have the potential to seriously limit personal aspirations” (Sucena et al., 2022, p. 2), which in the case of university students clearly include academic achievements.

Although the use of quantitative and qualitative methods for data collection and analysis in the present study can be considered as its strength, generalizing the present study results to large student populations is not possible due to its limitations such as the 7-week research process, small sample size and the limited number of scorers for evaluating student compositions. Consequently, due to the limited duration of the research process, it was not possible to provide training to the participants in terms of the use of computer-mediated learning environments or most recent AI tools for content-schemata activation in the study context. This can be undertaken in a longitudinal follow-up research. Despite these limitations, the present study contributes novel empirical data to the pertinent literature indicating that the use of skills activities prior to essay writing enables students to advance their cognition and to become better writers in the target language. In light of these positive findings, program designers are advised to integrate activities that activate ESL/EFL students’ content schemata into the curriculum of their language programs so that L2 practitioners can seamlessly use them to enable their students to connect their previous knowledge with new information which in turn leads to the enrichment of ideas in their L2 essays.

As students in tertiary level ESL/EFL education institutions are expected to not only participate in academic activities but also produce well-written academic English texts in terms of content and format, it is hoped that the results of this study will shed light onto endeavours towards the improvement of L2 writing in different ESL/EFL settings and inspire further research on the activation of content-schemata. Prospective studies might of course consider focusing on different aspects of schema theory such as comparing the impact of content-schemata activation with formal schemata. However, taking recent developments into consideration, future research should primarily investigate the integration of computer-mediated communication into L2 writing classes since “merely traditional teaching methods of writing skills are no longer appropriate” and we are living “in the age of fierce information technology development” (Anh, 2019, p. 82), which has without doubt become even more fierce after the breakout of the Covid-19 pandemic.

## References

[CR1] Anh DTN (2019). EFL students’ writing skills: Challenges and remedies. IQSR Journal of Research and Method in Education.

[CR2] Alisha F, Safitri N, Santoso I (2019). Students’ difficulties in writing EFL. Professional Journal of English Educator.

[CR3] Aron H (1986). Applying Shema theory to the teaching of reading. TESOL Newsletter.

[CR4] Bayat, N. (2014). The effect of the process writing approach on writing success and anxiety. *Educational Sciences: Theory & Practice, 14*(3), 1123–1141. 10.12738/estp.2014.3.1720

[CR5] Byrne D (1988). Teaching Writing Skills.

[CR6] Carell, P. & Eisterhold, J. (1988). Schema theory and ESL reading pedagogy. In Carell et al. *Interactive approaches to second language reading* (pp. 73–89). Cambridge: University Press.

[CR7] Chastain, K. (1976). *Developing second language skills: Theory and practice*. Harcourt Brade Jovanovich, Inc*.*

[CR8] Faraj AKA (2015). Scaffolding EFL students’ writing through the writing process approach. Journal of Education and Practice.

[CR9] Fareed M, Ashraf A, Bilal M (2016). ESL learners’ writing skills: Problems, Factors and Suggestions. Journal of Education and Social Sciences.

[CR10] Graham S, Perin D (2007). A meta-analysis of writing instruction for adolescent students. Journal of Educational Psychology.

[CR11] Graham S, Sandmel K (2011). The process writing approach: A meta-analysis. The Journal of Educational Research.

[CR12] Ibnian SSK (2017). Writing difficulties encountered by Jordanian EFL learners. Asian Journal of Humanities and Social Studies.

[CR13] Ivankova NV, Creswell JW, Stick SL (2006). Using mixed-methods sequential explanatory design: From theory to practice. Field Methods.

[CR14] Jouhar MR, Rupley WH (2021). The reading-writing connection based on independent reading and writing: A systematic review. Reading and Writing Quarterly.

[CR15] Li, Y., Nikitina, L., & Riget, P. N. (2022). Development of syntactic complexity in Chinese university students’ L2 argumentative writing. *Journal of English for Academic Purposes, 56*, 101099, 10.1016/j.jeap.2022.101099

[CR16] Maftoon P, Babamiri HM (2011). The effect of building formal schemata on EFL students’ writing achievement. TELL.

[CR17] Mahmood MHK, Nikoo FR, Bonyadi A (2013). The role of schema or background knowledge activation and graphic organizer on increasing Iranian EFL learners’ reading comprehension. European Online Journal of Natural and Social Sciences.

[CR18] Manzo U, Manzo A (2013). The informal reading-thinking inventory: Twenty-first-century assessment formats for discovering reading and writing needs-and strengths. Reading & Writing Quarterly.

[CR19] Norris S, Phillips L (1987). Explanations of reading comprehension: Schema theory and critical thinking theory. Teachers College Record.

[CR20] Oluwadiya, A. (1992). Some prewriting techniques for student writers. *English Teaching Forum, 30*(4)*,* 12–15*.*

[CR21] Perkins K, Angelis P (1985). Schematic concept formation: Concurrent validity for attained English as a second language reading comprehension. Language Learning.

[CR22] Qomariyah SSA, Permana D (2016). Process based approach towards students’ creativity in writing English paragraph. Indonesian Journal of English Language Teaching and Applied Linguistics.

[CR23] Sabuncuoğlu O (2018). A study of the effects of the approaches to the teaching of writing on the EFL instructors’ preferences at universities. RumeliDE Dil Ve Edebiyat Araştırmaları Dergisi.

[CR24] Selvaraj M, Aziz AA (2019). Systematic review: Approaches in teaching writing skill in ESL classrooms. International Journal of Academic Research in Progressive Education & Development.

[CR25] Shahriar, S., & Hayawi, K. (2023). *Let's have a chat! A Conversation with ChatGPT: Technology, Applications, and Limitations*. arXiv preprint arXiv:2302.13817.

[CR26] Spack R (1985). Literature, reading, writing, and ESL: Bridging the gaps. TESOL Quarterly.

[CR27] Statman S (1981). The activation of semantic memory: A pedagogical technique in the EFL classroom. ELT Journal.

[CR28] Sucena A, Silva AF, Marques C (2022). Reading skills intervention during Covid-19 pandemic. Humanities & Social Sciences Communications.

[CR29] Taglieber L, Johnson L, Yarbrough D (1988). Effects of pre-reading activities on EFL reading by Brazilian college students. TESOL Quarterly.

[CR30] Tahmourresi S, Papi M (2021). Future selves, enjoyment and anxiety as predictors of L2 writing achievement. Journal of Second Language Writing.

[CR31] Tavşanlı ÖF, Bilgin A, Yıldırım K, Rasinski T, Tschantz B (2020). The effect of a PBWMIP on writing success and attitude toward writing. Reading & Writing Quarterly.

[CR32] Thorne SL, Reinhardt J (2008). “Bridging activities”, new media literacies, and advanced foreign language proficiency. Calico Journal.

[CR33] Wood C, Schatschneider C (2021). Differential growth in writing quality of students in fifth grade from diverse backgrounds. Reading and Writing Quarterly.

[CR34] Zamel V (1987). Recent research on writing pedagogy. TESOL Quarterly.

[CR35] Zarrinabadi N, Rahimi S (2021). The effects of praise for effort versus praise for intelligence on psychological aspects of L2 writing among English-majoring university students. Reading & Writing Quarterly.

[CR36] Zhang ZV, Hyland K (2018). Student engagement with teacher and automated feedback on L2 writing. Assessing Writing.

